# Towards an Optimized Method of Olive Tree Crown Volume Measurement

**DOI:** 10.3390/s150203671

**Published:** 2015-02-04

**Authors:** Antonio Miranda-Fuentes, Jordi Llorens, Juan L. Gamarra-Diezma, Jesús A. Gil-Ribes, Emilio Gil

**Affiliations:** 1 Dpto. de Ingeniería Rural, Área de Mecanización y Tecnología Rural, Universidad de Córdoba, 14005 Córdoba, Spain; E-Mails: g62mifua@uco.es (A.M.-F.); ir2llcaj@uco.es (J.L.); o02gadij@uco.es (J.L.G.-D.); gilribes@uco.es (J.A.G.-R.); 2 Department of Agri Food Engineering and Biotechnology, Universitat Politècnica de Catalunya, Esteve Terradas 8, Campus del Baix Llobregat D4, 08860 Castelledfels, Barcelona, Spain

**Keywords:** terrestrial LiDAR, canopy characterization, olive tree, tree crown volume

## Abstract

Accurate crown characterization of large isolated olive trees is vital for adjusting spray doses in three-dimensional crop agriculture. Among the many methodologies available, laser sensors have proved to be the most reliable and accurate. However, their operation is time consuming and requires specialist knowledge and so a simpler crown characterization method is required. To this end, three methods were evaluated and compared with LiDAR measurements to determine their accuracy: Vertical Crown Projected Area method (VCPA), Ellipsoid Volume method (V_E_) and Tree Silhouette Volume method (V_TS_). Trials were performed in three different kinds of olive tree plantations: intensive, adapted one-trunked traditional and traditional. In total, 55 trees were characterized. Results show that all three methods are appropriate to estimate the crown volume, reaching high coefficients of determination: *R*^2^ = 0.783, 0.843 and 0.824 for VCPA, V_E_ and V_TS_, respectively. However, discrepancies arise when evaluating tree plantations separately, especially for traditional trees. Here, correlations between LiDAR volume and other parameters showed that the Mean Vector calculated for VCPA method showed the highest correlation for traditional trees, thus its use in traditional plantations is highly recommended.

## 1. Introduction

Increased awareness of the safe use of pesticides has led to substantial developments in the European environmental legal framework. Since the publication of the European Directive for a Sustainable Use of Pesticides in 2009 [1], great efforts have been made by all EU members to reduce the associated risks during the phase-use of pesticides. Of particular importance, is the need to establish procedures for identifying the most suitable dose and volume rate, especially in “three-dimensional” crops, such as orchards, vineyards, citrus and olive tree plantations. Establishing the most accurate volume rate for pesticide application in those crops appears to be one of the most difficult aspects, with most growers using a certain amount of subjectivity. There are several parameters that directly influence sprayer calibrations, and these are in turn, influenced by many external factors. In addition, uncontrollable factors such as weather conditions, pest and/or disease infestation, and crop development and its structure affect the final success of the spray application process.

Attempts to improve procedures to identify pesticide dose expression have included recommendations based upon either two (Leaf Wall Area: LWA) or three (Tree Row Volume: TRV) dimensional factors related to the canopy structure [2–4]. The high degree of variability in the crop canopy has hampered the development of general solutions to guarantee efficacy during the spraying process [5] and ensure that the most appropriate amount of pesticide is applied to all leaf surfaces with an even distribution, for crops of all types and in all situations.

According to that it is clear that precise measurements of external canopy dimensions leads to improved identification of pesticide dose. The chosen method for canopy characterisation, such as height and width is therefore of huge importance, and should be arranged by growers before spraying.

There are considerable differences between canopy characterization processes for a uniform canopy wall (*i.e.*, vineyard, orchards) and individual, isolated large trees, such as traditional olive tree plantations in the south of Europe. Olive tree plantations and olive oil production represent one of the most important incomes and activities in the agricultural sector on Mediterranean area with a total area of 7.7 Mha and a production over 11.6 MTm per year, and Spain is the largest producer of olive oil globally [6]. New alternative trellis systems have been adopted and developed for new olive tree plantations in recent years, to enable intensive farming. These produce increased tree density and a homogeneous canopy distribution along the row, but this represents only 2% of the olive cultivated area in Spain [7]. Further, traditional olive tree plantations represent 76% of total cultivated area, and intensive plantations represent 22% of cultivated area. Here, single, isolated and in most cases large, wide and heterogeneous canopy shapes can be identified. It is widely accepted that intensive orchards are more profitable than their traditional counterparts, due to the higher plantation density and the possibility of mechanical harvesting. As a result, there have been attempts to convert traditional plantations into intensive ones, by leaving one only trunk per tree, in order to allow the trunk shakers to harvest and plant new trees in between existing trees.

According to previous research, canopy measurement methods to characterise the whole tree structure can be classified in two groups: manual measurements and electronic procedures to estimate the most important tree dimensions. A range of manual methods for canopy characterisation has been widely applied to isolated trees. Among them, the ellipsoid method is the most widely used [8,9]. This method is impacted by the location of the measuring point selected for each tree, and so some authors propose to establish measurements at different heights of the canopy [10] to increase measurement precision.

Alternatively, the method of delimiting and measuring the projected area of the tree crown [11] has been proposed as a manual measurement process. Vertical crown projection onto the soil can be related to canopy volume [12]. Several possibilities for crown projection were established by the same authors, who proposed another canopy characterization methodology named *tree silhouette*. Tree canopy volume is estimated after applying the second theorem of Pappus Guldinus [13].

Electronic measurement methods use ultrasonic sensors and laser based sensors to estimate canopy characteristics. Ultrasonic sensors have been used for canopy volume measurements in vineyards [14–16], orchard fruits [4,17,18], and citrus plantations [10,19] due to its easy operation and management and affordable real-time data processing. However, there are doubts as to the accuracy of such measurements [20,21]. Further, laser technology has been found to achieve higher precision in comparison with ultrasonic sensors [14,19].

Laser technology is one of the most precise methods for canopy characterization [22] when applied to a range of crops using LiDAR 2D technology [22–26]. Furthermore, laser technology has been well implemented in olive tree canopies, where a complete characterization of the tree crown was achieved with a 3D laser scanner [27]. In this study, 24 trees belonging to four plots with a tree spacing of 7 × 7 m and 6 × 6 m (intensive disposition) were scanned from the top and one side of the crop. Excellent results were obtained for crown height, crown width, tree height, crown volume, and foliar density. Despite its precision, field management of those electronic devices is complex and not very well adapted to real field conditions where PPP must be applied. It may also be unrealistic to propose general implementation of these devices for wide use among the growers, due its complexity and cost. Conversely, accurate protocols for manual canopy characterization seem much more affordable and user-friendly, utilising simple and quick measurements. Whatever the selected method for canopy evaluation, it should guarantee some minimum requirements in terms of precision (as close as possible to the real canopy dimensions) in order to apply the most suitable amount of pesticide.

To this end, the aim of this research was to evaluate the accuracy of three different methods for manual canopy characterization (ellipsoid method, shade method and tree silhouette method) in traditional olive tree plantations, and to compare these with 2D LiDAR electronic measurements as a reference. Our objectives were:

(1) Define alternative manual canopy measurement protocol and compare it with electronic methods already in use.
(2) Evaluate the proposed methodologies in three different canopy types in olive tree plantations: intensive, adapted for mechanical harvesting, and traditional.
(3) Identify the most representative parameters for canopy characterization in olive trees.

## 2. Experimental Section

### 2.1. Characteristics of Selected Fields and Tree Plantations

**Figure 1.**
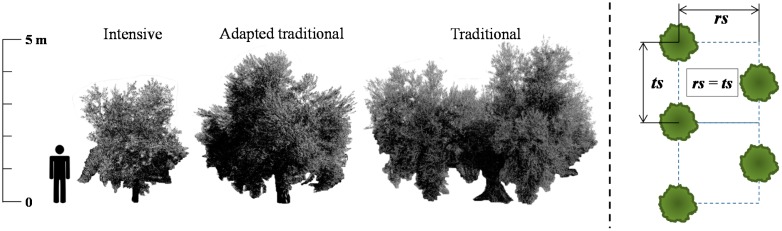
Olive tree types considered for the study (**left**) and the traditional trees distribution pattern (**right**).

Characterised trees were placed in two different fields, both of them located in the province of Córdoba (Andalusia, Spain), with the first one comprising two study plots: first field (37°45′46.78″N; 5°2′55.82″W) represents intensive and semi-intensive system, whilst the second field (37°43′8.43″N; 4°48′20.55″W) represented the traditional (several trunks) system. Plot numbers 1 and 3 present the *Picual* variety and plot 2 the *Gordal* variety (Table 1 and Figure 1) of olive trees. Plantation patterns consisted of square distributions (plot 1 and 2), and a quincunx distribution (plot 3). The selected culture systems are the most representative of the Spanish olive tree crop [7].

### 2.2. Manual Crown Measurement Methods Evaluated

Three different manual methods for crown measurement were selected: Vertical Crown Projected Area method (VCPA), Ellipsoid Volume method (V_E_), and the Tree Silhouette Volume method (V_TS_). Measurements corresponding to all the proposed methods and evaluated tree types were done the same day (Intensive: 25 February 2014, Adapted traditional: 19 March 2014, Traditional: 20 May 2014 and 2 July 2014) trying to avoid external and undesirable influences. A detailed explanation of the principles and procedure arranged for every one of the selected methods appears below. Those methods were compared with the results obtained with the electronic measurement method using LiDAR sensor.

#### 2.2.1. Vertical Crown Projected Area Method (VCPA)

**Figure 2.**
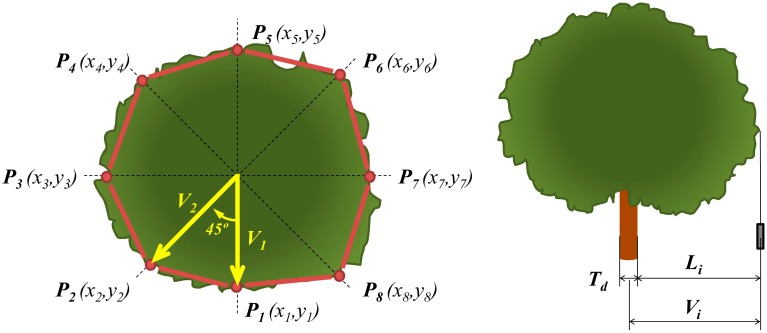
Vertical Tree Crown Projection's Mean Vector measurement method (**left**) and Tree crown projection measurement method (**right**).

The VCPA method is based on determining the projection of the tree crown onto the soil and determining its area, which can be correlated to its total volume. In order to do so, eight fixed directions (every 45 degrees) related to the north azimuth were selected for all the trees, (Figure 2) around the entire tree circumference. Vectors were measured from the centre of the trunk with a compass and a plummet placed in the most external point of the profile for each considered direction. If there was only one trunk, it was necessary to add half the Trunk Diameter (T_d_), which was obtained from the trunk circumference at 30 cm height. If there were two trunks, the origin of the vectors was set on the medium point between their centres, and if there were three, the origin was set on the barycentre of the triangle formed by the three. The Mean Vector parameter ( $\bar{MV}$) was then calculated as the mean of all the measured vectors (*V_i_*) according to Equation (1):

(1) $$\bar{MV}=\frac{\sum_{i=1}^{n}V_{i}}{n}$$

where $\bar{MV}$ is the Mean Vector Parameter (m); *V_i_* the single values of the eight measured vectors (m); and *n* the number of vectors for every single tree (8 vectors for a 45° space angling).

Taking into account the length and direction of every vector, it was possible to determine the coordinates of every single point acting as a vertex of the internal polygon (Figure 2) and, therefore, its area (A_PA_).

The internal polygon's area was calculated following Equation (2) corresponding to the Gauss's area algorithm, also known as shoelace method [23,28].

(2) $$A_{PA}=\frac{1}{2}\cdot \left|\sum_{i=1}^{n-1}x_{i}\cdot y_{i+1}+x_{n}\cdot y_{1}-\sum_{i=1}^{n-1}x_{i+1}\cdot y_{i}-x_{1}\cdot y_{n}\right|$$

where *x_i_* and *y_i_* are the coordinates of each point *i*.

#### 2.2.2. Ellipsoid Volume Method (V_E_)

Ellipsoid volume determination is based on assuming the tree crown to be an ellipsoid (defined by three semi axes) and determining its volume. Even though this method has been widely used in other studies [8,9,29], there is not a well-defined standard measurement protocol to obtain the required dimensions.

**Figure 3.**
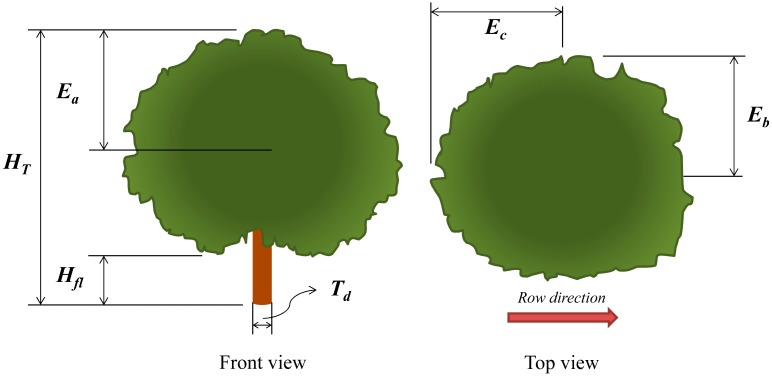
Parameters defined in the *Ellipsoid Volume* method.

In the present study, ellipsoid axes (E_a_, E_b_ and E_c_, as shown in Figure 3) were calculated using some of the vectors determined for the VCPA method. Therefore, semi axes E_b_ and E_c_ were calculated as the length of their corresponding vectors in North and East directions. In order to obtain semi axis E_a_, the total tree height (H_T_) and the height of the first leaf (H_fl_) were measured using a topographic milestone. E_a_ was calculated as the difference between H_T_ and H_fl_ and divided by 2. The final Ellipsoid Volume (V_E_) was calculated according Equation (3):

(3) $$V_{E}=\frac{4\pi }{3}\times E_{a}\times E_{b}\times E_{c}$$

#### 2.2.3. Tree Silhouette Volume Method (V_TS_)

The Tree Silhouette method (V_TS_) determines the crown volume by revolutionizing areas delimited on pictures taken from various positions around a vertical axis in the centre of the tree. Pictures were taken in the same orientation as the VCPA method, with a total of eight pictures per tree (P_i_). Pictures were scaled according to a reference (a topographic milestone set next to the tree) in the image processing software ImageJ^®^ (National Institutes of Health, Bethesda, MD, USA). Next, trunk position was determined and the tree canopy contour was manually delimited and automatically divided into two halves.

**Figure 4.**
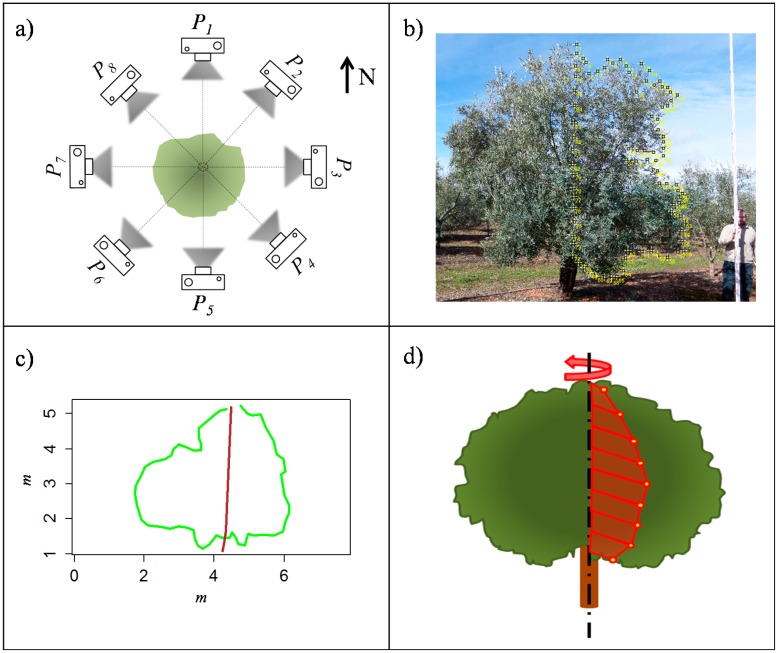
Procedure to characterise tree silhouette from each picture (P_i_) that was taken from all sides (every 45°) of the tree: (**a**) 8 Picture positions; (**b**) Manual contour delimitation; (**c**) Surface automatic calculation; and (**d**) Surface revolution.

A special program was developed in R software [30] to automatically calculate the surface of both of the crown projection halves and their respective volumes, by revolutionizing it around the vertical axis and using the Pappus Guldinus' second theorem. For each picture, the tree volume was calculated as the mean of the two generated volumes. The final volume of the tree crown was calculated as the mean of all the eight calculated volumes corresponding to the eight pictures (as shown in Figure 4).

### 2.3. LiDAR Canopy Characterization

**Figure 5.**
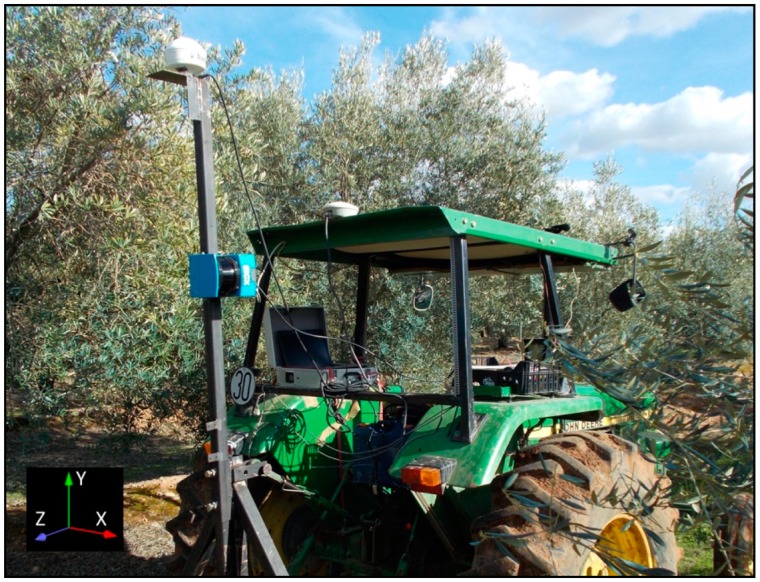
LiDAR sensor and laptop computer in the field, installed on the tractor.

A total of 55 trees randomly distributed on the selected parcels were scanned three times per side from the centre of the row, at a constant speed of 1 km·h^−1^. The LIDAR scanner used in this work was a low cost general-purpose model LMS-200 (Sick, Dusseldorf, Germany), with accuracy of ±10 mm and 5.2 mrad of divergence in a range up to 8 m, a selectable angular resolution of 1°, 0.5° or 0.25° and a scanning angle of 180°. The same device has been used previously [23,31]. It was mounted on a mast attached to a tractor and connected to a laptop via serial RS-232 port (as depicted in Figure 5). A GPS device AGGPS162 model (Trimble Navigation Ltd., Sunnyvale, CA, USA) with EGNOS correction was placed just above the sensor to determine absolute coordinates of the LiDAR points to relate the points obtained from each side of the tree. Two fixed references were used to correlate the GPS data to the LiDAR data, as described in Llorens *et al.* [23]. Even though the LiDAR sensor allows a maximum 0.25° resolution, speed limitations of the serial port communication meant that 1° resolution was chosen for the scans. However, this resolution is adequate for accurate characterisation of the canopy [22].

**Figure 6.**
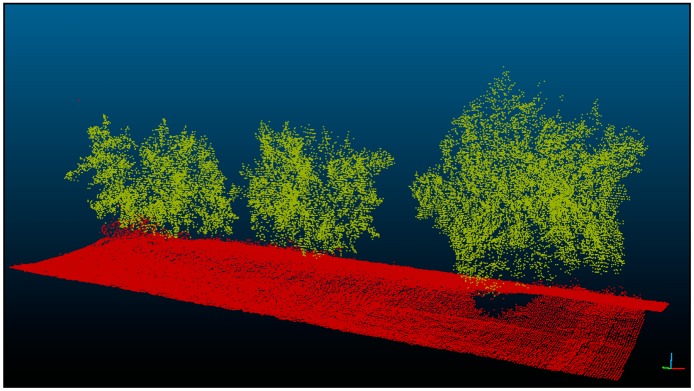
LiDAR cloud points obtained after scanning three contrasting trees on the same row. This cloud of points was used to estimate the tree volume.

LiDAR points and GPS coordinates obtained were processed to georeference each point obtained with the laser sensor. The total data files were filtered to discard those outside the crown (Figure 6).

**Figure 7.**
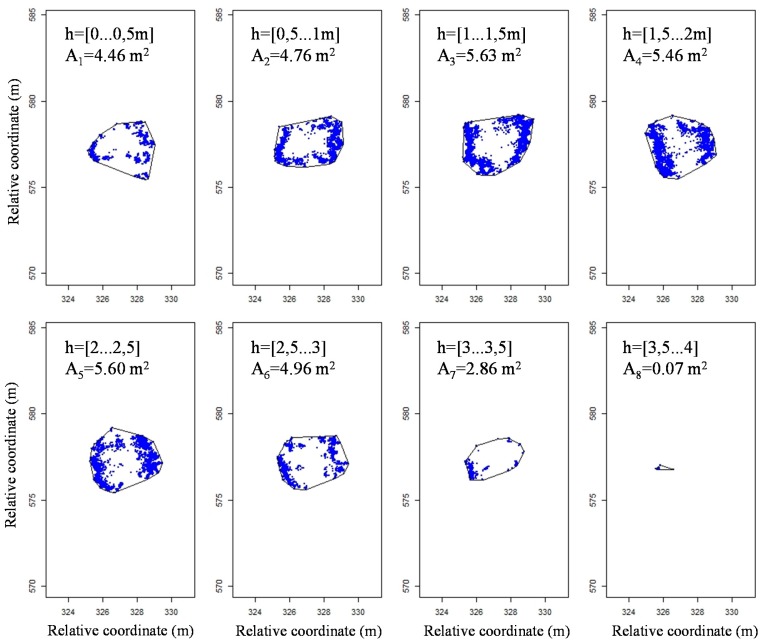
Projections of the points inside the different slices for a height interval of 0.5 m (Δh example for better representation of whole tree). Relative coordinates have been used.

A special program developed in R software was used to determine the volume represented by the point cloud. For this purpose, the method used by Xu *et al.* [12] was applied, which consists of calculating the crown volume by dividing the whole tree crown into different horizontal slices with the same height and aggregating all of its individual volumes. Data were classified into intervals of 0.01 m height each and represented on the same plane (Figure 7). The convex hull algorithm [32] was used to define a contour with the most external points of each single slice, and its area A_i_ was then calculated.

Finally, each area was multiplied for the height interval, giving an individual volume. The sum of every individual volume gave the total LiDAR tree crown volume (V_L_), as expressed in Equation (4)

(4) $$V_{L}=\sum_{1}^{n}\Delta h\times A_{i}$$

where *V_L_* is the calculated LiDAR volume in m^3^; Δ*h* is the height interval (m) and *A_i_* is the area inside the contour (m^2^).

### 2.4. Statistical Analysis

The statistical analysis adopted was a linear correlation between all measured and calculated parameters, using statistical R-Software with the *Agricolae* package. The data analysis included not only the results obtained by the method, but also the different geometrical parameters measured in order to determine any possible relationship with V_L_, assumed the most reliable and used as a reference volume. A Shapiro-Wilk test (*p* > 0.05) [33,34] and a visual inspection of their histograms, normal Q-Q plots and box plots were performed to ensure that the data were normally distributed for all the tree structures evaluated.

## 3. Results and Discussion

### 3.1. Geometrical Parameters of Evaluated Trees

Table 2 summarises all measured and calculated parameters. Canopies are observed to be large, with large trees ranging from 3.91 m in height in intensive plantations, up to 4.58 m in traditional culture systems. Such tree heights make crown characterisation difficult even with the LiDAR sensor, because the emitter is set to a constant height and thus the laser beam could not reach the upper part of the trees. Nevertheless, this is offset by the high row spacing, which allowed the sensor to be used with no data loss. Another important observation was the variability in trunk diameter amongst the plantations. Trunk diameter as measured in traditional trees was almost four times the magnitude and more variable (higher standard error) than those observed in intensive trees. This makes sense, due to the huge variety of different trunk shapes observed in the trees of traditional plantations.

We observe little variability in all shape parameters, *i.e.*, E_a_, E_b_, E_c_ and $\bar{MV}$, reaching 10 cm length in the most variable case. Relative errors are also small, ranging from 1.36% to 4.80%. This is especially true for $\bar{MV}$, due to its characterization of the whole shape of the crown.

Conversely, a large variability was observed in the estimated volume of various tree types amongst the methodologies evaluated. For example, considering V_L_, it ranges from 24.60 m^3^ for the intensive to 98.08 m^3^ for the traditional orchard, which supposes a four times increment of volume. Volume ranges obtained by all other methods were similar in magnitude. Variability within each tree structure was lower than 10% of the mean, even though standard errors were greater than 5 m^3^ in most traditional trees. Thus, accuracy of estimated volumes becomes less important with increasing tree volume, especially in traditional trees, which have the biggest sizes.

### 3.2. Comparison between LiDAR and Manual Methods for Tree Volume Estimation

**Figure 8.**
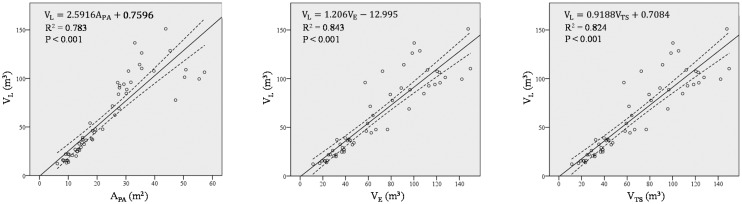
*R*^2^ values for linear correlations between LiDAR volumes (V_L_) and Vertical Projected Area (A_PA_) (**left**); Ellipsoid Volume (V_E_) (**centre**); and Tree Silhouette Volume (V_TS_) (**right**) for all orchard plantations. Dashed lines represent the 95% confidence interval for the mean.

Normality for all the tree types was confirmed by Shapiro-Wilk normality tests and visual inspection of their histograms, normal Q-Q plots and box plots. Therefore, data transformations were not deemed necessary. LiDAR tree crown volume (V_L_) was compared with volumes predicted by all other methods, as shown in Figure 8.

**Figure 9.**
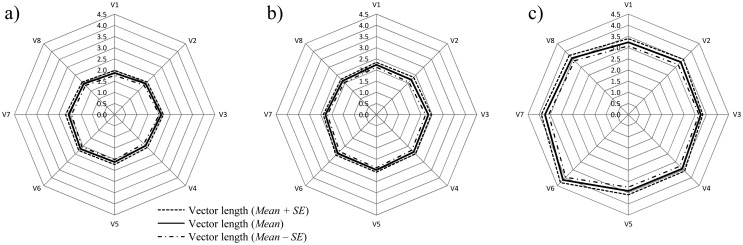
Mean Projected Area for each tree type: (**a**) Intensive; (**b**) Adapted Traditional and (**c**) Traditional. Graphical representation of Vertical Projected Area (A_PA_) parameter.

The three regression models demonstrate that the methods are in good agreement for the whole range of studied volumes (*R*^2^ = 0.79, *p* < 10^−3^ in all the cases). The Ellipsoid method (*R*^2^ = 0.84) performs best, in reference to the LiDAR measurements, and may therefore be considered the most appropriate method in olive tree volume characterisation after applying a multiplication factor of 1.2, according to the regression model (Figure 8). The Tree Silhouette method also fits the data with considerable accuracy (*R*^2^ = 0.824), and thus it may be a suitable alternative method. The Projected Area method demonstrated the lowest correlation (*R*^2^ = 0.785) with the LiDAR data. However, this method showed another important characteristic—the vector representation of the trees in all the eight directions identified the differential growth amongst the tree types (Figure 9). This is especially evident in traditional trees, where the mean Vertical Projected Area reflects a higher growth in a southwest direction.

We also observed that small tree volumes are more comparable with the V_L_ data, such as those in the intensive orchard, than for the larger trees belonging to traditional plantations. This could be accounted for by the irregularity of the traditional tree shapes, which are more easily measurable and characterised by the LiDAR sensor than by manual measurements. Electronic measurement by LiDAR easily detects the protruding branches and crown irregularities, which are common in biggest trees, and harder to characterise by any of the other proposed methods. In a decreasing order according their accuracy for irregularities' detection, methods can be ordered as: *Tree Silhouette Volume*, *Vertical Projected Area*, and *Ellipsoid Volume*.

Table 3 summarises the R^2^ values for the correlations between LiDAR tree crown volume (V_L_) and the other evaluated methods for each tree/plantation type. Tightest correlations between single-trunk trees, *i.e.*, intensive and adapted traditional trees and LiDAR estimates were observed, whilst Vertical Projected Area was the best estimator of LiDAR volume (*R*^2^ = 0.860) in orchards. Correlations of ellipsoid method (V_E_), and Tree Silhouette method (V_TS_) with LiDAR data, were very similar (*R*^2^ = 0.755 and *R*^2^ = 0.792, respectively).

In traditional adapted trees, the Tree Silhouette method presented the highest *R*^2^ value for its correlation with LiDAR data (*R*^2^ = 0.903), whilst the Ellipsoid method was the least correlated (*R*^2^ = 0.760). The highest *R*^2^ values were observed between LiDAR and all of the evaluated methods in the adapted crop system.

Finally, small positive determination coefficients were observed between the various measured parameters and LiDAR data for traditional tree shapes. As discussed earlier, this may be due to the reduction in measurement accuracy in the manual methods for high tree volumes (Figure 9). Even though not being very precise, the most accurate method for characterising traditional tree measurements among all the studied was found to be the ellipsoid method (*R*^2^ = 0.399), with the highest probability (*p* < 0.001) (*p* < 0.013 for A_PA_ and *p* < 0.007 for V_TS_).

### 3.3. Correlation between LiDAR Volume and Simple Canopy Parameters

We evaluated the use of various parameters used to calculate tree volume or area with LiDAR volume estimates, in order to simplify the field measurement methodology. Correlations of individual parameters with LiDAR volume are shown in Table 4. All measured parameters, except for the $\bar{MV}$ parameter, are weakly positively correlated with LiDAR volumes (*p* < 0.01). They are especially low for the Projected Area, Ellipsoid, and Tree Silhouette methods.

Only $\bar{MV}$ in the Projected Area method is strongly correlated with LiDAR volume (*R*^2^ = 0.903) (Table 4). Comparing this value with those obtained for the studied methods, it is found to be higher (highest R^2^ value was 0.843 for ellipsoid method, Figure 8). This is an important observation, as $\bar{MV}$ parameter calculation is a relatively simple and quick method that could be adopted by farmers or technicians without specialist training, in comparison with other methods such as electronic equipment is needed.

**Figure 10.**
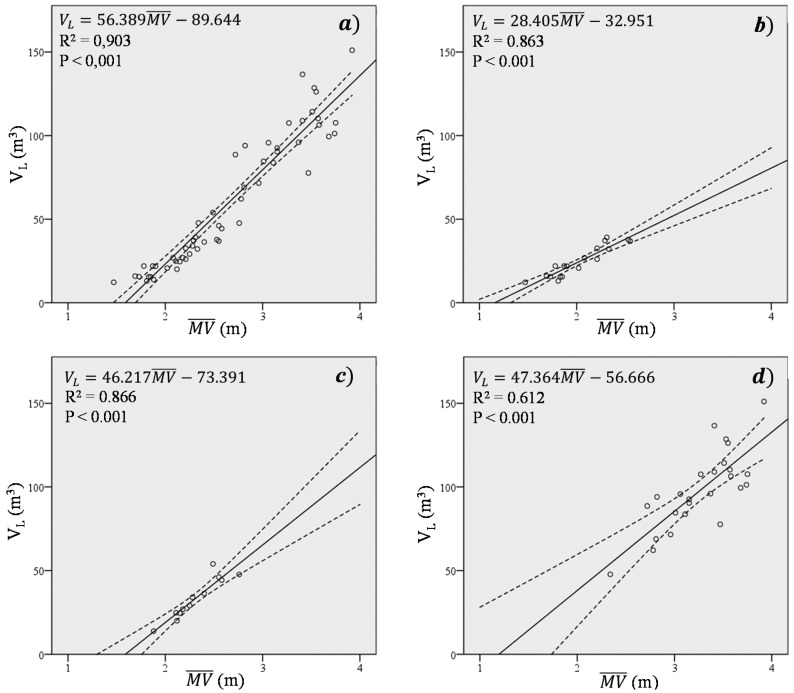
Correlations between $\bar{MV}$ and LiDAR volume for (**a**) All studied trees; (**b**) Intensive trees; (**c**) Adapted traditional trees; (**d**) Traditional trees.

The $\bar{MV}$ parameter correlates well with LiDAR volume across all plantation types, as shown in Figure 10. For example, determination coefficients are particularly high in intensive (*R*^2^ = 0.863) and adapted trees (*R*^2^ = 0.866). These coefficients of determination were as good as those obtained with the studied methods for these systems (Table 3). The most significant result was the coefficient of determination for traditional trees (*R*^2^ = 0.612), which was much higher than those similarly observed using the ellipsoid, tree silhouette and projected area methods. Thus, $\bar{MV}$ provides much more accurate estimations of crown volume than all other evaluated methods.

As to the relationships between each two of all the measured and calculated parameters, obtained correlations indicate proportionality in the trees' geometrical characteristics. Positive correlations are observed between H_T_ and $\bar{MV}$ (*R*^2^ = 0.450; *p* < 0.01) that leads to a relationship between crown width and height. Positive correlations are further observed between T_D_ and $\bar{MV}$ (*R*^2^ = 0.403; *p* < 0.01), semi axes E_b_ (*R*^2^ = 0.338; *p* < 0.01), and E_c_ (*R*^2^ = 0.413; *p* < 0.01). However, it must be underlined the low value of those correlations, which could suggest a certain dispersion of the values.

## 4. Conclusions

Three methodologies for measuring tree crown volumes were compared with those obtained with a 2D-LiDAR laser scanner in three types of olive tree plantations. The following conclusions can be drawn:

- All the evaluated methods were able to estimate the tree crown volume with a relatively high degree of accuracy. The best predictions were obtained with the Ellipsoid Volume measurement method, followed by the Tree Silhouette method and the Vertical Projected Area method.
- Correlations were not as good as those found in other three-dimensional crops due to the irregularity in the crown shapes. Determination coefficients were highest amongst low tree volumes, and weakest for high tree volumes.
- Vertical Projected Area method was the most accurate for intensive orchards, whilst the Tree Silhouette and Ellipsoid Volume method yielded the most accurate estimates of tree volume in adapted traditional orchards and traditional orchards, respectively. In traditional orchards, the coefficient of determination was much lower than in the adapted trees.
- Statistical analysis carried out demonstrated that all of the evaluated methods were able to estimate the crown volume in olive tree plantations, though new methodologies could be selected to achieve high accuracy.
- Study of different shape parameters showed that all the evaluated tree shapes have certain relationships between their basic dimensions, even though they are not very marked. This fact was mainly observed between tree height and crown width.
- Among all the parameters measured or calculated in this study, the Mean Vector used for the Vertical Projected Area method gave the best correlations amongst all trees in total, and for each individual tree shape. For traditional olive trees, the correlation of this parameter with the crown volume was much higher than those obtained with the other evaluated methods, but it is interesting to remark that the accuracy of the prediction is not as good as in the other crop types. In addition, the mean vector method seems to be a simple and quick procedure for canopy characterization, and requires no specialist training to be adopted. Therefore, it has been found to be the one of the most useful methods for estimating tree volume in traditional olive tree plantations. Nevertheless, its accuracy limitations should be considered.
- In general, whilst electronic LiDAR measurements was found to be the most accurate and reliable amongst all evaluated methods, alternative user-friendly methods (such as measurement of the $\bar{MV}$ parameter) could be proposed to characterize tree crown volume and dimensions (*i.e.*, shape). However, LiDAR measurement should not be considered as a perfect method. Problems linked to the measurement process itself [35] and collateral errors produced by GPS measurements [23] should be evaluated.

## Figures and Tables

**Table 1.** Main characteristics of parcel and trees selected for trials.

**Field Number**	**Plot Number**	**Tree Structure**	**Plantation Distances (rs × ts)**^1^	**N° of Trees Studied**
1	1	Intensive	7 m × 5 m	18
1	2	Traditional (1 trunk)	10 m × 12 m	12
2	3	Traditional (Several trunks)	12 m × 12 m ^2^	25

1 See Figure 1;

2 Plantation in quincunx structure.

**Table 2.** Mean and Standard Error of all parameters for each tree type.

	**Mean and Standard Error**		

	**Tree Type**		

	***Intensive***	***Adapted***	***Traditional***
**H_T_**(m)	3.91 ± 0.09	4.52 ± 0.11	4.58 ± 0.05
**T_D_**(m)	0.19 ± 0.01	0.41 ± 0.04	0.75 ± 0.08
**E_a_**(m)	1.82 ± 0.05	2.09 ± 0.06	2.10 ± 0.03
**E_b_**(m)	2.07 ± 0.09	2.33 ± 0.09	3.24 ± 0.08
**E_c_**(m)	1.96 ± 0.09	2.25 ± 0.07	3.07 ± 0.10
$\bar{\text{MV}}\;(\text{m})$	2.03 ± 0.07	2.31 ± 0.07	3.27 ± 0.08
**A_PA_(m^2^)**	11.88 ± 0.83	15.39 ± 0.96	35.58 ± 2.06
**V_E_(m^3^)**	31.90 ± 3.00	46.74 ± 3.52	87.64 ± 3.79
**V_TS_(m^3^)**	29.69 ± 2.32	45.11 ± 3.92	100.26 ± 5.21
**V_L_(m^3^)**	24.60 ± 2.19	33.49 ± 3.58	98.08 ± 5.21

**Table 3.** *R*^2^ values for correlations between LiDAR tree crown volume (V_L_) and the other evaluated methods for each tree structure.

	**Intensive**	**Adapted Traditional**	**Traditional**

	**V_L_** (m^3^)		
**A_PA_(m^2^)**	0.860 **	0.835 **	0.242 *
**V_E_(m^3^)**	0.755 **	0.760 **	0.399 **
**V_TS_(m^3^)**	0.792 **	0.903 **	0.275 **

* for *p* < 0.05 and

** for *p* < 0.01

**Table 4.** Multiple correlations between different parameters obtained in the study for all the tree types.

	**H_T_(m)**	**T_D_(m)**	$\bar{\text{MV}}\;(\text{m})$	**E_a_(m)**	**E_b_(m)**	**E_c_(m)**	**V_L_(m^3^)**
**H_T_** (m)	1	0.129 **	0.450 **	0.912 **	0.326 **	0.309 **	0.419 **
**T_D_** (m)		1	0.403 **	0.123 **	0.338 **	0.413 **	0.342 **
$\bar{\text{MV}}\;(\text{m})$			1	0.402 **	0.664 **	0.748 **	0.903 **
**E_a_** (m)				1	0.294 **	0.299 **	0.353 **
**E_b_** (m)					1	0.423 **	0.676 **
**E_c_** (m)						1	0.654 **
**V_L_** (m^3^)							1

* for *p* < 0.05 and

** for *p* < 0.01.
